# Facebook Ads Manager as a Recruitment Tool for a Health and Safety Survey of Farm Mothers: Pilot Study

**DOI:** 10.2196/19022

**Published:** 2021-04-07

**Authors:** Richard R Burke, Bryan P Weichelt, Kang Namkoong

**Affiliations:** 1 National Farm Medicine Center Marshfield Clinic Research Institute Marshfield, WI United States; 2 Department of Communications University of Maryland College Park, MD United States

**Keywords:** Facebook, recruitment, advertisement, agriculture, health, safety, survey, online

## Abstract

**Background:**

Social media platforms have experienced unprecedented levels of growth and usage over the past decade, with Facebook hosting 2.7 billion active users worldwide, including over 200 million users in the United States. Facebook users have been underutilized and understudied by the academic community as a resource for participant recruitment.

**Objective:**

We performed a pilot study to explore the efficacy and cost-effectiveness of Facebook advertisements for the recruitment of an online agricultural health and safety survey.

**Methods:**

We undertook a 1-week advertising campaign utilizing the integrated, targeted advertising platform of Facebook Ads Manager with a target-spending limit of US $294. We created and posted three advertisements depicting varying levels of agricultural safety adoption leading to a brief survey on farm demographics and safety attitudes. We targeted our advertisements toward farm mothers aged 21-50 years in the United States and determined cost-effectiveness and potential biases. No participant incentive was offered.

**Results:**

We reached 40,024 users and gathered 318 advertisement clicks. Twenty-nine participants consented to the survey with 24 completions. Including personnel costs, the cost per completed survey was US $17.42. Compared to the distribution of female producers in the United States, our advertisements were unexpectedly overrepresented in the eastern United States and were underrepresented in the western United States.

**Conclusions:**

Facebook Ads Manager represents a potentially cost-effective and timely method to recruit participants for online health and safety research when targeting a specific population. However, social media recruitment mirrors traditional recruitment methods in its limitations, exhibiting geographic, response, and self-selection biases that need to be addressed.

## Introduction

Researchers are facing a range of issues in recruiting participants, including decreasing response rates and increased difficulties in reaching participants through traditional modes such as phone and paper [1-3]. In turn, these challenges are associated with increased recruitment costs. As the presence of social media has increased and as our communication strategies have shifted, researchers are increasingly looking to these systems for potential recruitment to address current challenges.

Facebook is the largest social media platform with over 1.8 billion daily active users and 2.7 billion monthly active users [4]. Of these, over 200 million users are within the United States. This high proportion of users allows Facebook advertising to reach potential research participants in a short period of time. As of 2019, up to 90% of US adults use the internet, a number that has been steadily growing [5]. Additionally, although 24% of Americans do not have an internet connection at home, 17% of total US adults use a smartphone without home internet access, thereby increasing the feasibility of internet-based research [6].

One of the largest barriers in recruitment is cost. Mailed survey costs are relatively evenly distributed, ranging from US $6.51 to $30.24; however, this includes high personnel costs [7-9]. Telephone surveys have been observed to range from US $29 to $99 per participant, also requiring high personnel costs [10,11]. Some online health and behavioral studies reported costs as low as US $0.64 per participant, whereas others reported costs up to US $33 per participant; however, the median cost tends to be skewed toward the lower end of the scale [12-15].

Both telephone and mailed surveys require preexisting information to reach a targeted population, whereas Facebook’s integrated Ads Manager utilizes its internal user data to determine such demographics. However, this broad targeting introduces further concerns over multiple biases [13,16]. Although more traditional recruitment methods have been used and evaluated for agricultural populations, often with mixed results, our review of the literature indicates that there has been little data reported on the use of Facebook or other social media platforms for recruitment [17-21]. Therefore, we undertook this pilot study to investigate the effectiveness of targeted advertisements on Facebook as a tool to recruit participants in the agricultural sector, and to assess possible self-selection and geographic biases in this sampling method.

## Methods

To determine the feasibility and cost-effectiveness of Facebook advertisements for an agricultural health and safety survey, we ran an advertising campaign consisting of three separate advertisements, each with a different agricultural image. The campaign was performed on Facebook from March 9, 2020 to March 16, 2020 at a total cost of US $294, distributed evenly among the three advertisements. Images were selected based on the apparent safety adoption displayed to determine if advertisement imagery affected performance. This included low (overturned tractor), neutral (child feeding a calf), and high (woman sweeping a barn in full personal protective equipment) safety adoption (see Multimedia Appendicies 1-3 for examples of advertising images). These advertisements were run concurrently through the National Children’s Center for Rural and Agricultural Health and Safety (NCCRAHS) Facebook page. We investigated the cost per click, cost per participant, and potential geographical biases that arose during this campaign.

We chose the following inclusion criteria to target our advertisements: individuals currently living in the United States, identified as female, within the ages of 21-50 years, and had a “role,” as defined by Facebook, in Farming, Fishing, or Forestry (FFF; delineated by Facebook as Demographics > Work > Industries > FFF). Facebook reported 170,000 eligible users according to these criteria. This campaign directed users who clicked on the advertisement to an online, REDCap-hosted consent document outlining a novel open 10-item survey assessing farm family demographics, views on child agricultural safety, and open-ended feedback on the Facebook advertisement they were shown [22,23]. This survey was devised in anticipation of a research project assessing the impacts of relevant news media on farm mothers’ knowledge, attitudes, and behavioral intentions toward childhood agricultural safety. No financial incentive was provided for participation. The survey instrument, consent form, and protocol for this study were deemed exempt from review by the Marshfield Clinic Research Institute Institutional Review Board.

We investigated the geographic distribution of advertisements by comparing Facebook’s advertisement targeting system performance to an expected distribution based on the number of female producers at the state level [24]. We then mapped the ratio of expected to observed reach of the advertisements.

## Results

Over this 1-week period, the total reach of the advertising campaign (measured by number of views) was 40,024 and 318 users clicked on one of the advertisements, representing a click rate of 0.79% (Table 1). Of these 318 visitors, 29 consented to the survey, representing a participation rate of 9.1%, with 24 fully completing the survey for a completion rate of 83%. This yields a completed response rate of 0.06% out of the total reach and 7.5% out of the total clicks. The total cost per click for this study was US $0.92, whereas the cost per participant was US $10.14. With the 17% dropout rate (completed surveys vs consenting participants) and a total personnel cost of US $31 per hour at 4 hours, the total cost per completed survey was US $17.42.

The neutral advertisement depicting a child feeding a calf generated the most clicks with 122 out of 318 total clicks (38.4%). However, the image of a young woman sweeping the barn had the highest click rate at 0.84% (Table 1).

**Figure 1:**
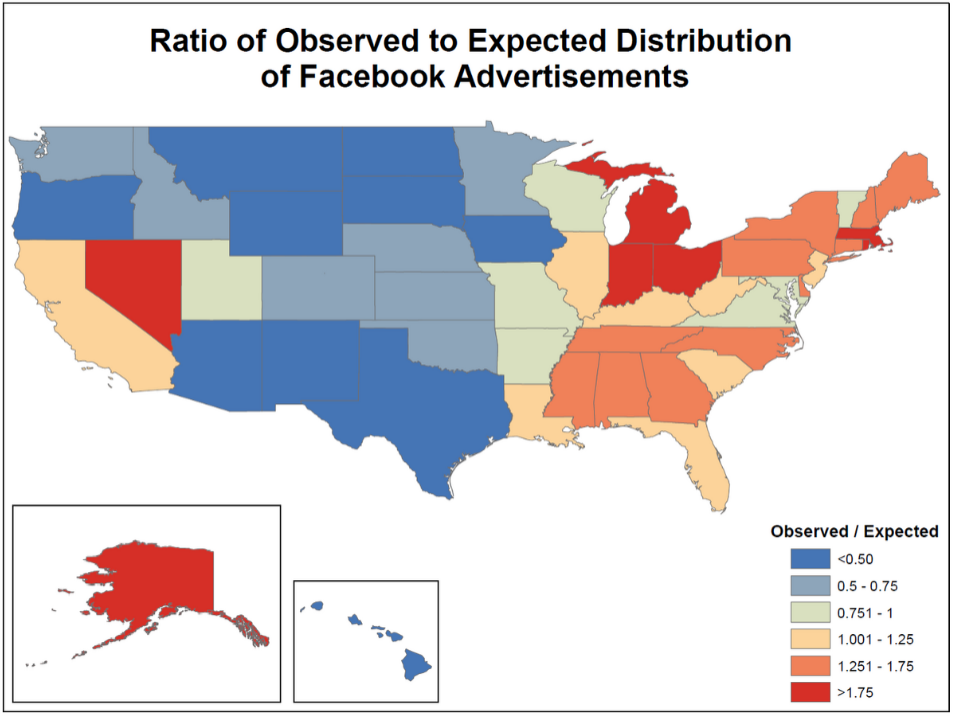
Ratio of observed to expected geographical distribution of Facebook ads. Data obtained from the United States National Agricultural Statistics Service 2017 Census of Agriculture.

Advertisements were posted more often than expected in eastern states, whereas western states (excluding California, Nevada, and Utah) tended to be overrepresented compared to the distribution of female producers (Figure 1). There was no observed difference in geographical distribution among the three advertising images.

**Table 1:** Reach and click rate of advertisement images.

Advertisement image	Reach (N)	Click rate, n (%)
Overturned tractor	14,240	102 (0.72)
Child feeding calf	14,652	122 (0.83)
Woman sweeping	11,132	94 (0.84)
Total	40,024	318 (0.79)

## Discussion

### Principal Findings

This pilot project aimed to determine if Facebook Ads Manager could be a useful tool for recruiting samples of the agricultural population for online health and safety surveys. The current adoption of Facebook and other social media in the United States, as well as increasing rates of internet access, show promise for future research to be performed in part or entirely online.

We reached over 40,000 Facebook users at a cost of US $294 and generated a total cost per completed survey of US $17.42. This is comparable to other observed costs ranging between US $0.64 and $33 for online health and behavioral studies; however, we were unable to identify any published studies that utilized Facebook Ads Manager for recruiting an agricultural population such as farmers, ranchers, farm or ranch parents, or farmworkers specifically [12-15]. It should be noted that as advertisements approach the total sampling frame for a study, there is the risk of reaching saturation among the target population, meaning that individuals will be shown the same advertisement multiple times. Further research is needed to determine what diminishing returns, if any, exist when recruiting larger sample sizes and showing a single advertisement multiple times to the same people.

### Limitations

This pilot study had several limitations. First, our study had a remarkably small final response rate of 0.06% based on total reached (N= 40,024) and of 7.5% based on total clicks (N=318). Facebook and other online methods of recruitment allow for high scalability with relatively low cost. However, with this low response rate, it is possible for other, larger samples to reach a level of saturation among the target population, resulting in advertisements being shown multiple times to potential participants.

Additionally, this study exhibited a level of geographic bias that was algorithmically determined by the Facebook Ads Manager platform. Facebook users across multiple states were shown the advertisement a disproportionately high or low amount when compared to the expected target population residing in those states. Although online studies allow for ease in reaching national and international samples, the risk of bias remains prominent, and appropriate measures should be taken to adjust or account for these discrepancies (eg, data weighting, stratification, quota sampling).

We did not offer or disburse any financial incentive for participation. Although we had 318 visits to the survey, only 29 visitors consented to participate and 24 fully completed the survey. We were also unable to accurately measure response rates and completion rates for each advertisement image. Future studies should ensure that the survey infrastructure is set up to measure and assess these potential differences. This campaign was administered via the NCCRAHS Facebook Page, which then appeared as the host organization ads, and may have influenced which respondents engaged with the advertisements, in turn affecting click rates and completion rates. The provision of compensation could help increase the participation and completion rates; however, measures should be taken to control for the additional self-selection bias such measures could introduce.

It should be noted that we do not have full control over, or knowledge of, Facebook’s mechanisms for targeted advertising. The woman sweeping advertisement had lower reach than the other images, as determined by Facebook, but had the highest click rate. Additionally, Facebook reported 170,000 women, aged 21-50 years, who have a role in FFF. The National Agricultural Statistics Service 2017 Census estimated over 500,000 female agricultural producers between the ages of 21 and 50 years in the United States; this does not include farm hands or women whose spouse works in agriculture [24].

### Future Work

We argue for additional research to make use of these relatively underutilized resources alongside sufficient measures to control for geographic and response biases, as well as misrepresentation and data validity. Future research should include data weighting, verifying consistent responses across similar questions, and “insider knowledge” questions [25,26]. More in-depth studies and analyses could offer insight into additional methods for applying these methods to online survey studies and how these methods may differ for agricultural populations.

### Conclusion

We were successful in using Facebook Ads Manager to recruit a sample of an agricultural population for an online health and safety survey in a short time frame. We observed low response rates, which were counteracted by a large advertisement reach. This method shares many limitations with traditional survey recruitment methods and requires appropriate measures to limit potential biases. Future research is needed to determine the effectiveness of Facebook Ads Manager on recruiting a large agricultural population and if this method is sustainable.

## Appendix

Multimedia Appendix 1 Preview of low safety adoption advertisement.

Multimedia Appendix 2 Preview of neutral safety adoption advertisement.

Multimedia Appendix 3 Preview of high safety adoption advertisement.

## Abbreviations

FFF: Farming, Fishing, and Forestry
NCCRAHS: National Children’s Center for Rural and Agricultural Health and Safety
