# Supplementary figures and images for: Facebook Ads Manager as a Recruitment Tool for a Health and Safety Survey of Farm Mothers: Pilot Study
Source: JMIR Form Res. 2021 Apr 7;5(4):e19022. doi: 10.2196/19022 (PMC8060866; doi:10.2196/19022)

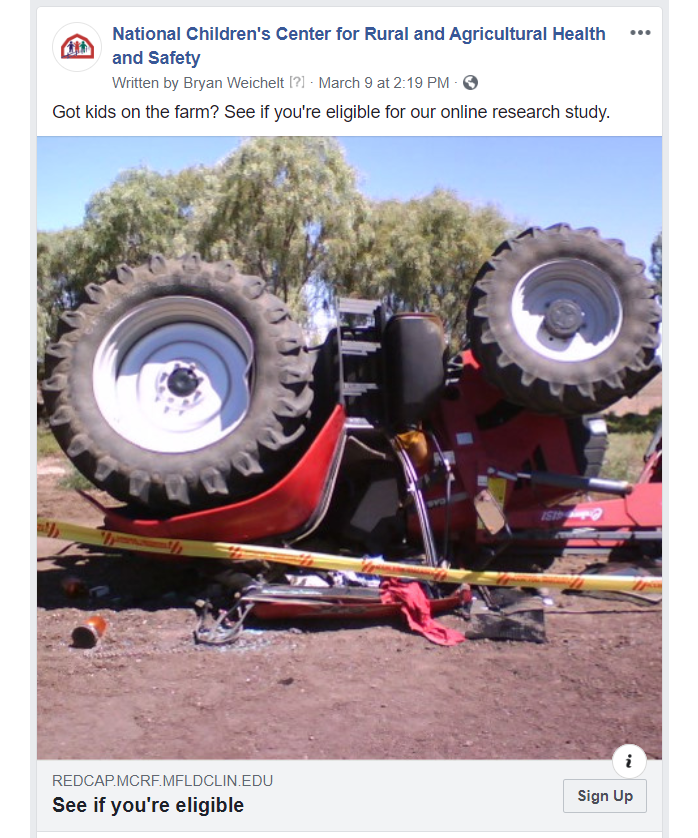

Supplement: Multimedia Appendix 1 [file formative_v5i4e19022_app1.png]

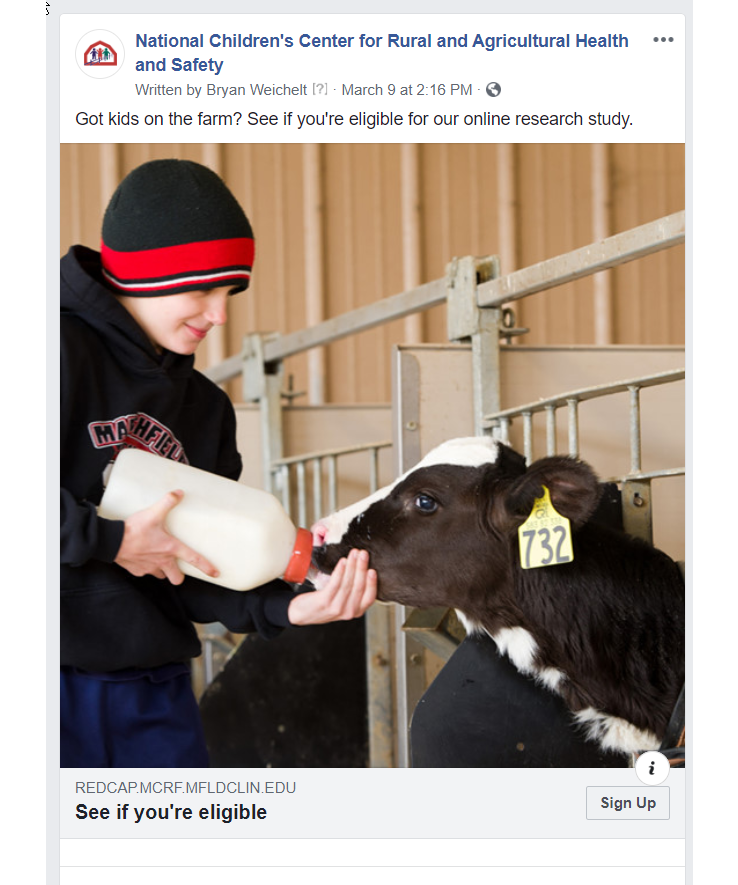

Supplement: Multimedia Appendix 2 [file formative_v5i4e19022_app2.png]

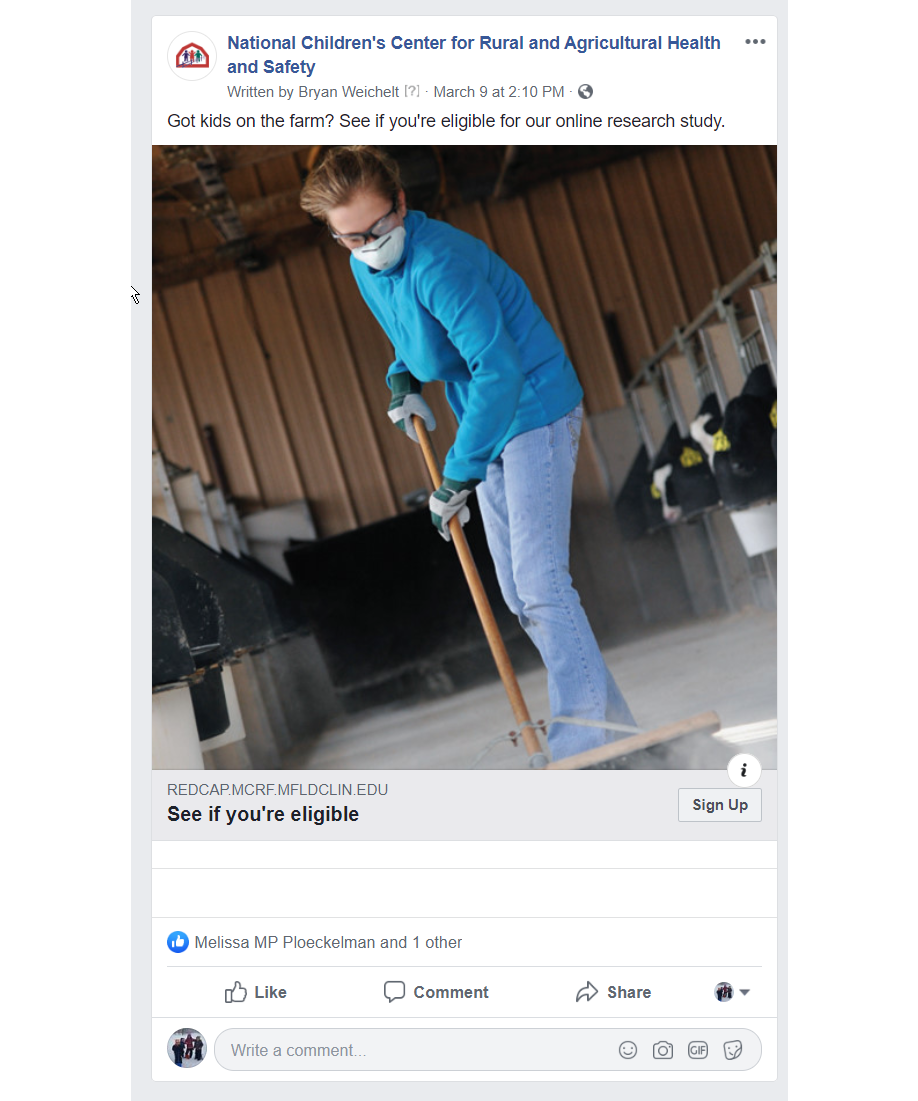

Supplement: Multimedia Appendix 3 [file formative_v5i4e19022_app3.png]
